# Intracorporeal Versus Extracorporeal Colo-colic Anastomosis in Minimally-invasive Left Colectomy: a Systematic Review and Meta-analysis

**DOI:** 10.1007/s11605-023-05827-1

**Published:** 2023-09-12

**Authors:** Sascha Vaghiri, Dimitrios Prassas, Sarah Krieg, Wolfram Trudo Knoefel, Andreas Krieg

**Affiliations:** 1 Department of Surgery (A), Heinrich-Heine-University and University Hospital Duesseldorf, Moorenstr. 5, Bldg. 12.46, 40225 Duesseldorf, Germany; 2 Clinic for Gastroenterology, Hepatology and Infectious Diseases, Heinrich-Heine-University and University Hospital Duesseldorf, Duesseldorf, Germany

**Keywords:** Intracorporeal anastomosis, Extracorporeal anastomosis, Left colectomy, Outcome

## Abstract

**Purpose:**

The primary aim was to investigate the operative outcomes of intracorporeal (IA) and extracorporeal (EA) anastomosis in left-sided minimally-invasive colectomy.

**Methods:**

A comprehensive literature search was conducted for studies comparing operative outcomes and follow-up data of IA versus EA in minimally-invasive left colectomy. Studies that investigated recto-sigmoid resections using transanal circular staplers were excluded. Data from eligible studies were extracted, qualitatively assessed, and included in a meta-analysis. Odds ratios (ORs) and mean differences with 95 per cent confidence intervals were calculated.

**Results:**

Eight studies with a total of 750 patients were included (IA *n* = 335 versus EA *n* = 415). IA was associated with significantly lower overall morbidity (OR 0.40, 95% CI 0.26–0.61, *p* < 0.0001) and less frequent surgical site infection (SSI) (OR 0.27, 95% CI 0.12–0.61, *p* = 0.002) as primary outcomes compared to EA. Of the secondary outcomes, length of incision (SMD -2.51, 95% CI -4.21 to -0.81, *p* = 0.004), time to first oral diet intake (SMD -0.49, 95% CI -0.76 to -0.22, *p* = 0. 0004) and time to first bowel movement (SMD -0.40, 95% CI -0.71 to -0.09, *p* = 0.01) were significantly in favor of IA, while operative time was significantly shorter in the EA group (SMD 0.36, 95% CI 0.14–0.59, *p* = 0.001).

**Conclusions:**

IA proves to be a safe and feasible option as it demonstrates benefits in terms of lower overall morbidity, fewer rates of SSI, smaller incision length, and faster postoperative gastrointestinal recovery despite a longer operative time compared to EA.

**Supplementary Information:**

The online version contains supplementary material available at 10.1007/s11605-023-05827-1.

## Introduction

Minimally-invasive colorectal surgery for benign and malignant diseases has become the gold standard over the past decades since its introduction in 1991.^1^ Many high quality studies have demonstrated not only equal technical feasibility and safety compared to the open surgery, but also a sustained benefit and superiority in terms of short-term outcomes, postoperative recovery including earlier resumption of oral diet and less postoperative pain, length of hospital stay, and improved quality of life.^2–7^ On the other hand long-term oncologic outcomes were comparable between both approaches.^8,9^ After the successful transition to minimally-invasive procedures the focus has now shifted to technical modifications in laparoscopic and robotic surgery in order to optimize processes and reduce operative related morbidities. Two types of anastomosis have been implemented in minimally-invasive colorectal surgery: intracorporeal anastomosis (IA) and extracorporeal anastomosis (EA). Both techniques have advantages as well as disadvantages. During IA procedure the bowel is opened inside the abdomen which increases the risk of bowel content contamination and tumor cell spillage. Conversely EA requires extensive bowel mobilization and exteriorization which exerts traction on the mesentery and may result in postoperative trauma and impaired bowel motility.^10–12^ The surgical results of IA and EA methods in right colectomy have been extensively compared in many randomized and non-randomized studies. IA demonstrated significantly better cumulative results for the following outcome parameters: overall complications, time to first bowel movement, postoperative pain, length of incision, surgical site infection, anastomotic leak, conversion rate, and incisional hernia.^13–15^ Of note, in robotic right colectomy, IA was associated with a significantly longer operative time as compared to EA,^16^ while this difference was less pronounced in laparoscopic procedures.^13^ Given the broad indications for left-sided colectomy, ranging from colorectal cancer to diverticular disease, few studies have compared the advantages and disadvantages of IA versus EA in minimally- invasive left-sided colectomy, as this procedure poses more technical challenges to surgeons than right-sided colectomy.^17,18^ Therefore, the aim of this study was to perform a meta-analysis of studies comparing the feasibility and safety of IA and EA techniques in patients undergoing minimally-invasive left hemicolectomy for benign and malignant indications with special emphasis on short- term outcomes.

## Material and Methods

The meta-analysis was conducted according to the current Preferred Reporting Items for Systematic Reviews and Meta-Analyses (PRISMA) checklist^19^ and the Cochrane Handbook for Systematic Reviews of Interventions.^20^

### Search Strategy

A systematic database search was conducted in Pubmed (Medline), and google scholar, and the Cochrane Central trials register without time or language restrictions. The following key search terms were used in combination with the Boolean operators AND or OR: "extracorporeal", "intracorporeal", "colectomy" and "anastomosis". In addition, the reference list of the retrieved studies was screened to identify potentially relevant citations for the analysis. Two reviewers (S.V. and D.P.) independently assessed each selected abstract and study for eligibility and inclusion in the meta-analysis. Disagreements were resolved either by consensus or by consultation with a third author (S.K.) when necessary. The last literature search was performed on June 1^st^, 2023.

### Eligibility Criteria

All original studies comparing the outcomes of IA and EA in minimally-invasive left-sided colectomy were included, regardless of sample size. As this meta-analysis focused on the anastomotic techniques performed, IA was the intervention of interest compared to the extracorporeal approach (comparator). Patients with both malignant and non-malignant pathology located from the transverse colon to the proximal third of the sigmoid colon undergoing either laparoscopic or robotic left colectomy with a colo-colic anastomosis were included. We excluded studies with sigmoid or (anterior) rectum resection and a transanal end-to-end mechanical double stabled colorectal anastomosis due to the difference of the applied anastomotic techniques and thus comparability. To be included in the meta-analysis, studies had to report on at least one of the following procedure-related outcomes: intraoperative morbidity, postoperative complications, operative time, and recovery parameters. Non-comparative studies and articles that included both left- and right-sided colectomies without subgroup analysis of IA/EA procedures in left colectomy were excluded. In case of duplicate or overlapping articles published by the same institution and authors, the most recent study was selected for inclusion.

### Data Extraction and Outcome Measures

A self-developed electronic data extraction sheet was used independently and blindly by two authors (S.V., D.P.) to enter all relevant data, if complete, from studies meeting the eligibility criteria. Disagreements were discussed and resolved by consensus or reassessment by a third author (S.K.). The following data were retrieved from each included study:

1. General study characteristics: first author, year and country of origin, study design, enrollment period, number of patients enrolled in each group, inclusion and exclusion criteria, follow-up period, study endpoints
2. Demographics: Age, sex, BMI (body mass index), ASA (American Society of Anesthesiologists) classification, previous abdominal surgery or pelvic radiation, indication for surgery (benign or malignant), colonic location of pathology, TNM stage
3. Technical aspects and operative characteristics: type of access (robotic, laparoscopic, laparoscopic-assisted), type and configuration of anastomosis, number of trocars and ports used, operative time, length of incision, site of specimen extraction, duration of surgery, conversion rate, estimated blood loss, use of indocyanine green (ICG), and number of harvested lymph nodes
4. Postoperative complications: anastomotic leak, intra-abdominal fluid/abscess collection, anastomotic bleeding, postoperative transfusion, surgical site infection (SSI), postoperative ileus, cardiac and pulmonary events, mortality, 30-day reoperation rates, and incisional hernia
5. Postoperative recovery data: postoperative pain assessed by visual analogue scale (VAS) on days 0 and 3, time to first postoperative flatus, time to first postoperative bowel movement, time to first oral diet intake, and length of hospital stay

The primary outcomes of this study were overall postoperative morbidity, severe postoperative morbidity (Clavien-Dindo > III),^21^ anastomotic leak, anastomotic bleeding, surgical site infection, intra-abdominal fluid/abscess collection, postoperative transfusion, postoperative ileus, and reoperation rates. The secondary outcomes of interest were operative time, length of incision, number of harvested lymph nodes, specimen length, resection margin, blood loss, conversion to laparotomy, and postoperative recovery data: VAS on days 0 and 3, time to first oral diet intake, time to first flatus, time to first bowel movement, and length of hospital stay.

### Quality Assessment

The risk of bias of the included non-randomized trials was independently assessed by two authors (S.V. and D.P.) using the ROBINS-I tool.^22^ It consists of 7 different domains of bias at 3 time points in each study: Pre-intervention (confounding and selection of participants), at intervention (classification of interventions), and post-intervention (bias due to deviations from the intended interventions, missing data, measurement of outcomes, and selection of the reported outcome). Based on these criteria, the risk of bias in each study is categorized as “low”, “moderate”, “serious “, and “critical”. The investigators were not blinded to the study authors. Disagreements in grading were discussed and resolved by consensus or reassessment by a third author (S.K.). The Grading of Recommendations, Assessment, Development, and Evaluation (GRADE) method^23,24^ with 4 assigned levels of evidence (high, moderate, low, and very low) was used to adequately document the strength of evidence for the significant outcomes.

### Statistical Analyses

Statistical analysis was performed using RevMan software (version 5.3. Copenhagen: The Nordic Cochrane Centre, The Cochrane Collaboration, 2014) according to the recommendations of the Cochrane Collaboration guidelines. Pairwise meta-analyses were performed. For each endpoint of interest, summary treatment effect estimates with 95% confidence intervals (CIs) were calculated. For dichotomous outcomes, odds ratios (ORs) and the Mantel–Haenszel method were used. Standardized mean differences (SMDs) were calculated to analyze continuous outcomes. The methods proposed by Hozo et al.^25^ and Luo et al.^26^ were applied to convert available median and interquartile range (IQR) data to mean and standard deviation for continuous variables. The level of heterogeneity among the included studies was interpreted as follows after using the Cochrane’s Q test (Chi-squared test; Chi^2^) and measuring inconsistency (I^2^): 0%-40% low heterogeneity and might not be important, 30%-60% moderate heterogeneity, 50%-90% substantial heterogeneity, > 75% high heterogeneity.^20,27^ Note that starting with moderate heterogeneity, the significance of the obtained I^2^ value is dependent on the size and direction of the effects and the power of evidence for heterogeneity (e.g., p-value of the Chi-squared test or the I^2^ confidence interval).^20^ If heterogeneity was low or moderate (I^2^ < 50%), summary estimates were calculated using a fixed-effects method. Otherwise, if I^2^ > 50%, the random-effects model was used. In cases of substantial heterogeneity, the source of heterogeneity was further investigated using one-way sensitivity and subgroup analyses. Subgroup analyses were performed according to study size (≥ median sample size versus < median sample size), study bias (low versus moderate-high bias), single-center versus multi-center design, study origin and propensity score matching (PSM) to test the stability of the meta-analysis. Publication bias tests and funnel plots were not performed because of the small number of studies included in the meta-analysis. *p*-values < 0.05 of pooled data were considered significant.

## Results

**Fig. 1:**
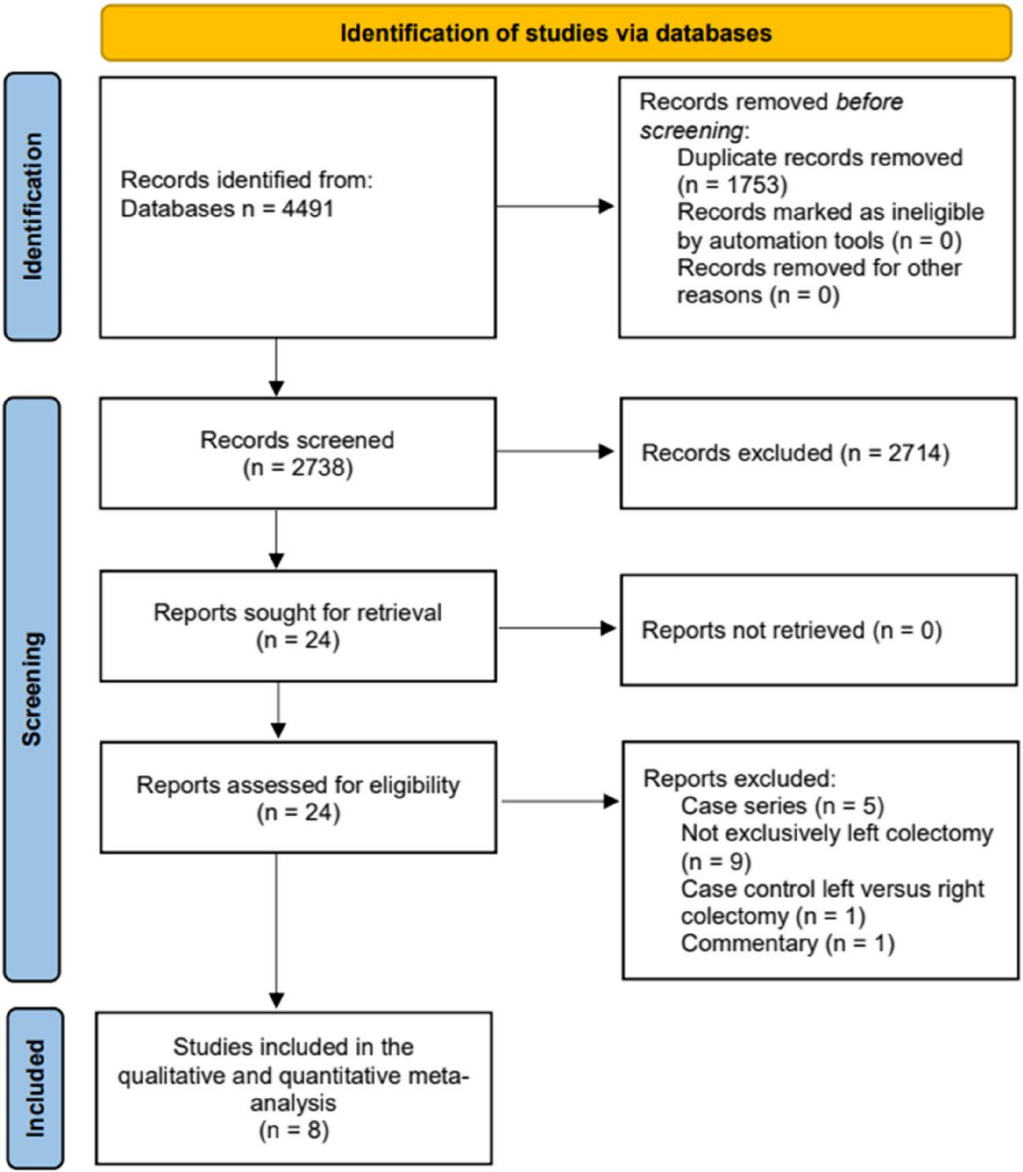
PRISMA diagram of study identification and selection for review analysis

The study selection flowchart is shown in Fig. 1. The initial electronic database search identified 4491 articles, of which 8 studies^28–35^ met the inclusion criteria and were eligible for the final meta-analysis.

### Study and Patient characteristics

A total of 750 patients (IA: 335 cases versus EA: 415 cases) originating from 5 different countries (Italy, Japan, Taiwan, China and Israel) undergoing minimally-invasive left colectomy were included in 8 observational studies.^28–35^ The study enrolment period was from January 2004 to September 2021. The study by Teramura et al.^35^ included a subgroup analysis of 43 patients with left-sided colectomy among a total of 283 cases with both left-and right sided colectomies. The male to female ratio was 436:271 in 7 studies^28–34^ with available demographic data for IA and EA patients undergoing left colectomy. Except for 3 studies^30,31,34^ with a multi-center design, 5 studies^28,29,32,33,35^ were single-center studies. The operative indication was malignant tumors in 7 studies^28–34^ while Teramura et al.^35^ also included benign diseases. Laparoscopic and laparoscopic-assisted left colectomy was performed in all studies while robotic approaches were not reported. The site of pathology was located in the transverse colon/splenic flexure in 55.33% and descending colon/proximal third of sigmoid colon in 44.67%. In both IA and EA groups, the majority of anastomosis were stapled (89.05%), while hand-sewn anastomosis was performed in only 10.95%. One study did not report the proportion of stapled and hand-sewn anastomosis.^28^ Follow-up ranged from 1 to 95 months. The complete study and patient characteristics are summarized in Tables 1 and 2, while the technical aspects of the studies are presented in Table 3.

**Table 1:** Study characteristics

Author	Year	Origin	Study design	PSM	Study period	Total sample size	Type of access	Site of pathology (*n*)	Operative indication		Follow-up period
									Malignant (%)	Benign (%)	
Carlini et al.^28^	2016	Italy	Single-center, prospective	no	Jan 2004-Oct 2015	20	Laparoscopic-assisted totally laparoscopic	Splenic flexure 20	100	0	1–95 months
Swaid et al.^29^	2016	Israel	Single-center, retrospective	no	Jan 2005-Sept 2014	52	Laparoscopic-assisted, totally laparoscopic	Distal transverse colon 10 Splenic flexure 5 Descending colon 37	100	0	30-days
Milone et al.^30^	2018	Italy	Multi-center, prospective	no	Jan 2005-Dec 2015	181	Laparoscopic-assisted, totally laparoscopic	Splenic flexure 181	100	0	30-days
Grieco et al.^31^	2019	Italy	Multi-center, retrospective	yes	Jan 2008-Aug 2017	72	Laparoscopic	Splenic flexure 72	100	0	30-days
Masubuchi et al.^32^	2019	Japan	Single-center, retrospective	yes	May 2013-Dec 2017	40	Laparoscopic	Left-sided colon 40 (distal descending colon-proximal sigmoid colon)	100	0	NA
Wang et al.^33^	2022	Taiwan	Single-center, retrospective	no	Jul 2016-Sep 2019	117	Laparoscopic	Transverse colon 39 Splenic flexure 13 Descending colon 65	100	0	1–24 months
Guo et al.^34^	2023	China	Multi-center, retrospective	yes	Jan 2015-Sep 2021	225	Laparoscopic-assisted Laparoscopic	Distal transverse colon/ Splenic flexure 47 Descending colon 64 Sigmoid colon (prox. third) 114	100	0	30-days
Teramura et al.^35^ *	2023	Japan	Single-center, retrospective	yes	Jan 2018-Jun 2021	43	Laparoscopic	Transverse colon 28 Descending colon 15	NA	NA	30-days

*PSM* Propensity score matching; *NA* Not available, * subgroup analysis left colectomy

**Table 2:** General patient characteristics

Author	Groups	No. of patients	Gender (M/F)	Age (years) mean ± SD	BMI (Kg/m^2)^ mean ± SD	ASA score	Previous abdominal surgery	Previous pelvic radiation	Pathology	
									T4	N pos
Carlini et al.^28^	IA/EA	20	13/7	68.4 ± 7.2	25 ± 3.8	ASA I/II 18 ASAIII 2	5	NA	6	12
Swaid et al.^29^	TLC/IA	33	22/11	64 ± 12.4	25.4 ± 3.9	ASA I/II 26 ASA III 7	NA	NA	None	NA
	LAC/EA	19	8/11	72.7 ± 2.1	25 ± 3.6	ASA I/II 12 ASA III 7	NA	NA	None	NA
Milone et al.^30^	TLC/IA	92	54/38	66 ± 10.9	29.5 ± 4.3	ASA I/II 51 ASA III/IV 41	29	NA	8	37
	LAC/EA	89	47/42	68.7 ± 10.24	24.7 ± 4.2	ASA I/II 51 ASA III/IV 38	30	NA	10	27
Grieco et al.^31^	IA	36	19/17	71.4 ± 9.9	25.3 ± 4.0	ASA I/II 25 ASA III 11	11	NA	2	13
	EA	36	23/13	68.7 ± 6.7	26.0 ± 4.5	ASA I/II 28 ASA III 8	16	NA	4	18
Masubuchi et al.^32^	IA	20	11/9	63.25 ± 12.5	26.95 ± 6.15	ASA I 8 ASA II 12	3	NA	3	4
	EA	20	14/6	63 ± 14.75	22.475 ± 2.575	ASA I 6 ASA II 14	2	NA	4	3
Wang et al.^33^	IA	40	23/17	61.45 ± 11.9	23.92 ± 3.1	ASA II 15 ASA III/IV 25	6	NA	1	10
	EA	77	45/32	62.65 ± 13.5	23.94 ± 4.6	ASA II 34 ASA III/IV 43	18	NA	15	27
Guo et al.^34^	TLLC/IA	84	58/26	61.64 ± 11.31	23.9 ± 2.79	ASA I-II 80 ASA III-IV 4	NA	NA	67*	40
	LALC/EA	141	99/42	61.35 ± 11.23	24.02 ± 3.67	ASA I-II 131 ASA III-IV 5	NA	NA	116*	65
Teramura et al.^35^ **	IA	21	NA	NA	NA	NA	NA	NA	NA	NA
	EA	22	NA	NA	NA	NA	NA	NA	NA	NA

*ASA score* American Society of Anesthesiologists; *BMI* Body max index; *SD* Standard deviation; *CICA* Completely intracorporeal anastomosis; *ECAA* Extracorporeal assisted anastomosis; *LALC* Laparoscopic-assisted left colectomy; *LAC* Laparoscopic-assisted colectomy; *TLC* Totally laparoscopic; *IA* Intracorporeal anastomosis; *EA* Extracorporeal anastomosis; *NA* Not available, *pT3-4, **data subgroup left colectomy missing

**Table 3:** Technical description

Author	Groups	No. of patients	Type of anastomosis	Use of ICG	Number of trocars/ports	Site of extraction
Carlini et al.^28^	IA	9	side-to-side, antiperistaltic, stapled 9	no	3–4	Pfannenstiel
	EA	11	side-to-side, isoperistaltic, hand-sewn 11	no	3–4	Off-midline
Swaid et al.^29^	TLC/IA	33	side-to-side, isoperistaltic, stapled 33	no	4	Mini-Pfannenstiel
	LAC/EA	19	side-to-side, isoperistaltic, stapled 19	no	4	Left off-midline
Milone et al.^30^	TLC/IA	92	side-to side, stapled 82 end-to-end, hand-sewn 10	no	NA	Mini-Pfannenstiel
	LAC/EA	89	side-to side, stapled 85 end-to-end, hand-sewn 4	no	NA	Mini-laparotomy midline
Grieco et al.^31^	IA	36	side-to-side, isoperistaltic, stapled 36	no	3–4	Pfannenstiel
	EA	36	isoperistaltic, stapled 15 or hand-sewn 21	no	3–5	Left subcostal
Masubuchi et al.^32^	IA	20	side-to-side, isoperistaltic, stapled 20	no	5–6	Midline
	EA	20	side-to-side, antipersitaltic, stapled 20	no	5–6	Midline
Wang et al.^33^	IA	40	end-to-end, hand-sewn side-to-side, isoperistaltic, stapled side-to-side, antiperistaltic, stapled	no	4	Pfannenstiel, midline, natural orifice specimen extraction, off-midline
	EA	77	side-to-side, antiperistaltic, stapled end-to-end, isoperistaltic, hand-sewn	no	4	Midline, umbilical wound
Guo et al.^34^	TLLC/IA	84	side-to-side, stapled 83, handswen 1	no	NA	Longitudinal midline, off-midline
	LALC/EA	141***	end-to end, handswen 20 side-to-end, stapled 94 end-to-side, stapled 6	no	NA	Longitudinal midline, off-midline
Teramura et al.^35^	IA	21	side-to-side, isoperistaltic,stapled 21	no	5	Pfannenstiel, umbilical midline
	EA	22	side-to-side, iso-and antiperistaltic, stapled 22	no	5	Umbilical midline

*CICA* Completely intracorporeal anastomosis; *ECAA* Extracorporeal assisted anastomosis; *LALC* Laparoscopic-assisted left colectomy; *LAC* Laparoscopic-assisted colectomy; *TLC* Totally laparoscopic; *IA* Intracorporeal anastomosis; *EA* Extracorporeal anastomosis; *ICG* Indocyanine green; *NA* Not available, *** 21 cases missing after PSM

### Primary Outcome Analysis

#### Statistically Significant Primary Outcomes

##### Overall Morbidity

**Fig. 2:**
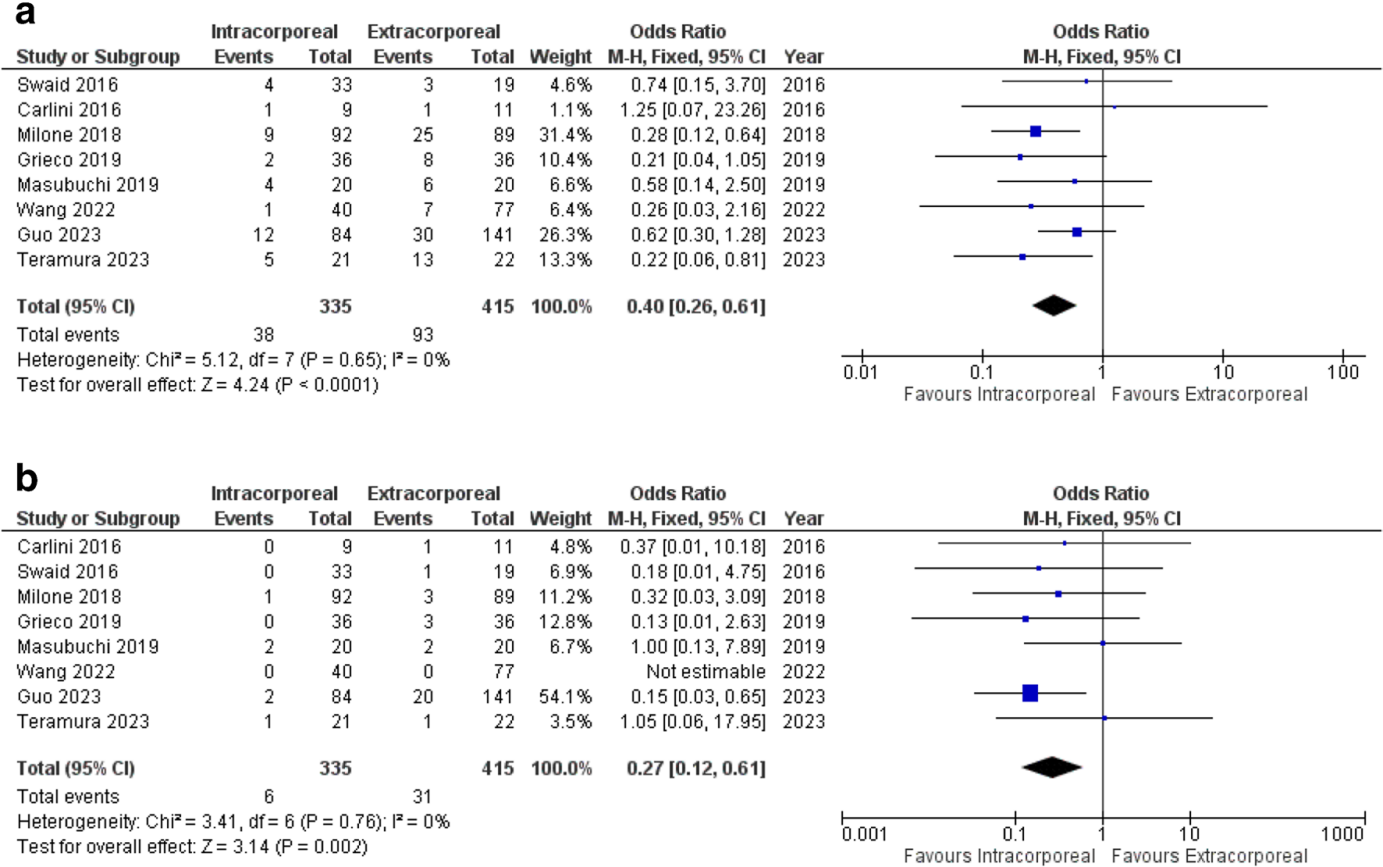
Forest plots of significant primary outcomes (IA versus EA): **(a)** overall morbidity; (**b)** SSI

Overall morbidity was reported in all 8^28–35^ included studies. Meta-analysis of the pooled data revealed a significantly higher incidence of overall complications in the EA group compared to the IA cohort (OR 0.40, 95% CI 0.26–0.61, *p* < 0.0001). Notably, the level of heterogeneity was low (I^2^ = 0%, Chi^2^ test: *p* = 0.65) (Fig. 2a).

##### Surgical Site Infection (SSI)

The SSI rate was reported in 8 studies^28–35^ including 750 cases. Patients with IA had a significantly lower rate of SSI compared to patients with EA (OR 0.27, 95% CI 0.12–0.61, *p* = 0.002). The degree of heterogeneity was low (I^2^ = 0%, Chi^2^ test: *p* = 0.76) (Fig. 2b).

#### Statistically Non-significant Primary Outcomes

Non-significant differences between minimally-invasive IA and EA were observed for the following primary outcomes: severe postoperative morbidity (Clavien-Dindo > III), intra-abdominal fluid/abscess collection, anastomotic leak, anastomotic bleeding, postoperative transfusion, postoperative ileus, and reoperation rates. (Table 4).

**Table 4:** Non-significant primary and secondary outcomes

Outcomes	No. of included studies	No. of included patients		SMD/OR [95% CI]	*p*-value	Heterogeneity level	
		IA	EA			I^2^ (%)	Chi^2^ (*p*-value)
Primary							
Severe complications *	6^28,30,31,33–35^	282	376	0.66 [0.30–1.46]	0.30	36	0.18
Intra-abdominal fluid/abscess	8^28–35^	335	415	0.69 [0.26–1.82]	0.45	0	0.79
Anastomotic leak	8^28–35^	335	415	0.61 [0.24–1.56]	0.30	0	0.83
Anastomotic bleeding	8^28–35^	335	415	3.14 [0.81–12.21]	0.10	0	0.97
Postoperative transfusion	4^28–31^	170	155	0.84 [0.19–3.81]	0.83	0	0.65
Postoperative ileus	6^28–30,32,33,35^	215	238	0.33 [0.08–1.40]	0.13	0	0.63
Reoperation rate	5^28–31,33^	210	232	0.87 [0.23–3.24]	0.83	0	0.73
Secondary							
Number of harvest lymph nodes	4^30,31,33,34^	252	343	−0.03 [−0.25–0.19]	0.78	39	0.18
Specimen length (cm)	2^31,33^	76	113	−0.07 [−0.36–0.23]	0.66	0	0.50
Resection margin (cm)	2^33,34^	124	218	0.18 [−0.04–0.40]	0.11	0	0.60
Blood loss (ml)	2^32,33^	60	97	−0.38 [−0.86–0.10]	0.12	44	0.18
Postoperative pain day 0	2^32,33^	60	97	0.04 [−0.49–0.58]	0.88	55	0.14
Postoperative pain day 3	2^32,33^	60	97	−0.13 [−0.45–0.20]	0.44	0	0.56
Time to first flatus	5^29–33^	221	241	−0.25 [−0.59–0.09]	0.15	65	0.02
Length of hospital stay	7^28–33,35^	251	274	−0.40 [−0.84–0.03]	0.07	80	< 0.0001

*OR* Odds ratio; *SMD* Standardized mean difference; *IA* Intracorporeal anastomosis; *EA* Extracorporeal anastomosis, * Clavien-Dindo > III

### Secondary Outcome Analysis

#### Statistically Significant Secondary Outcomes

##### Length of Incision

**Fig. 3:**
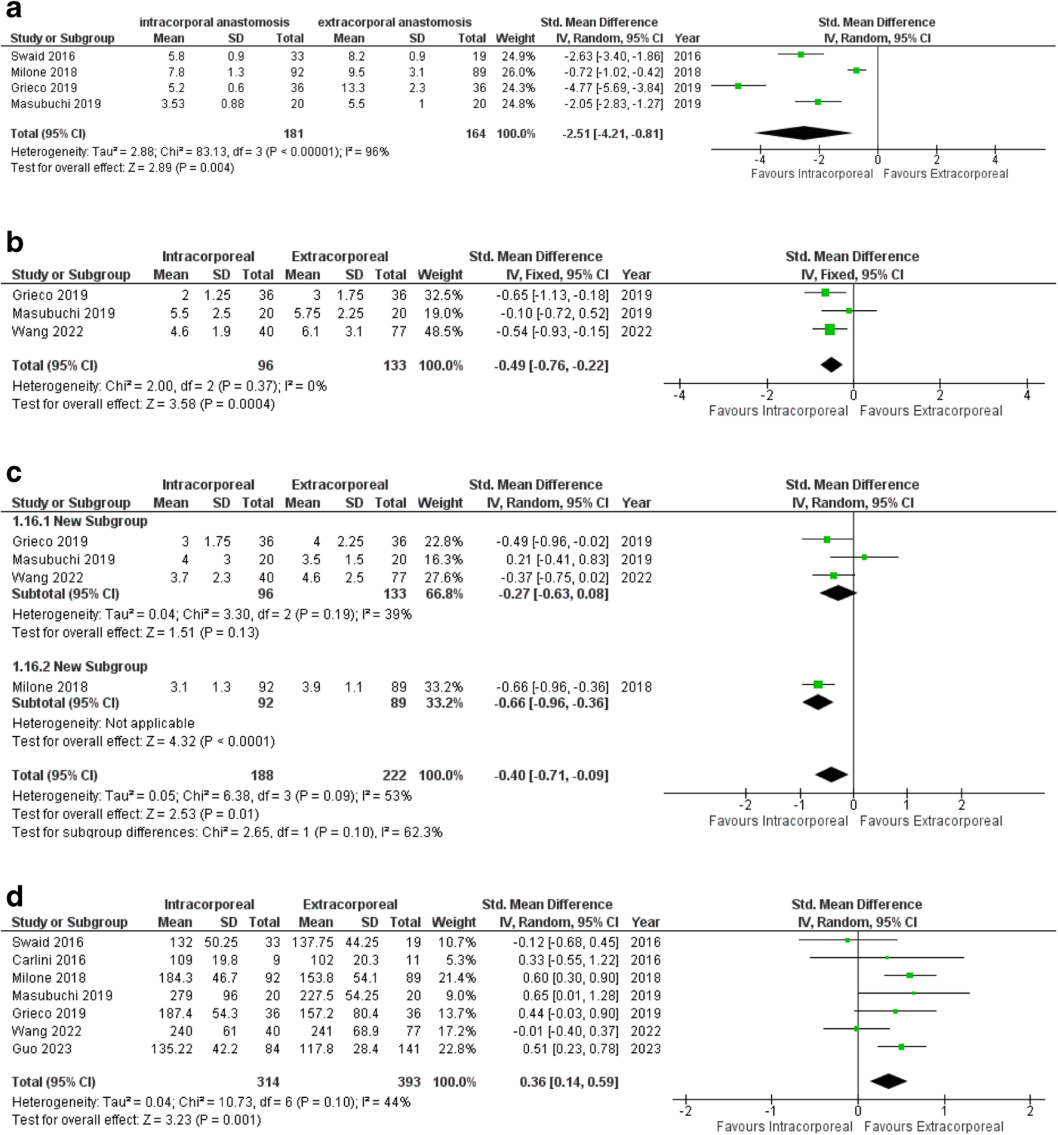
Forest plots of significant secondary outcomes (IA versus EA): **(a)** length of incision; (**b)** time to first oral diet intake; (**c)** time to first stool passage; **(d)** operative time

The length of incision was reported in 4 studies^29–32^ with 345 patients. IA was associated with a significantly shorter incision length compared to EA (SMD -2.51, 95% CI -4.21 to -0.81, *p* = 0.004) (Fig. 3a). Importantly, the heterogeneity between studies was very high (I^2^ = 96%, Chi^2^ test: *p* < 0.00001). However, neither the one-way sensitivity analysis nor the subgroup analysis could identify a single study or a specific factor that could explain the large heterogeneity.

##### Time to Oral Diet Intake

Three studies^31–33^ with 229 patients were included in the meta-analysis of time to first reported postoperative oral diet. The IA group had a significantly shorter time to first oral diet intake as compared to the EA group (SMD -0.49, 95% CI -0.76 to -0.22, *p* = 0.0004) (Fig. 3b). A low level of heterogeneity was observed (I^2^ = 0%, Chi^2^ test: *p* = 0.37).

##### Time to First Stool Passage

Four studies^30–33^ reported the time of first bowel movement including 410 patients. The meta-analysis showed a significantly faster return of bowel movement in the IA group compared to the EA group (SMD -0.40, 95% CI -0.71 to -0.09, *p* = 0.01) (Fig. 3c). The degree of heterogeneity was substantial (I^2^ = 53%, Chi^2^ test: *p* = 0.09). One-way sensitivity analysis identified the study published by Milone et al.^30^ as the source of heterogeneity.

##### Operative Time

The duration of surgery was reported in 7 studies.^28–34^ Minimally-invasive left colectomy performing IA was associated with a significant longer operative time compared to EA (SMD 0.36, 95% CI 0.14–0.59, *p* = 0.001) (Fig. 3d). The level of heterogeneity was moderate (I^2^ = 44%, Chi^2^ test: *p* = 0.10).

#### Statistically Non-significant Secondary Outcomes

Meta-analysis of the secondary outcomes of interest revealed no statistically significant difference between the IA and EA groups in number of harvested lymph nodes, specimen length, resection margin, blood loss, postoperative pain on days 0 and 3, time to first flatus, and length of hospital stay (Table 4).

However, the secondary outcomes showed at least substantial heterogeneity for postoperative pain on day 0, time to first flatus, and length of hospital stay. For postoperative pain on day 0, no one-way sensitivity or subgroup analysis was performed because only 2 studies^32,33^ analyzed this outcome. For length of hospital stay, only studies with a cohort ≥ 62 patients^30,31,33^ demonstrated a shorter length of stay in the IA group. In addition, heterogeneity was less evident (I^2^ = 55%, Chi^2^ test: *p* = 0.11) in this subgroup, suggesting that study size may be the cause of heterogeneity. For other subgroups, this difference was neither confirmed nor heterogeneity reduced (Table S1).

### Quality and Risk of Bias

**Fig.4:**
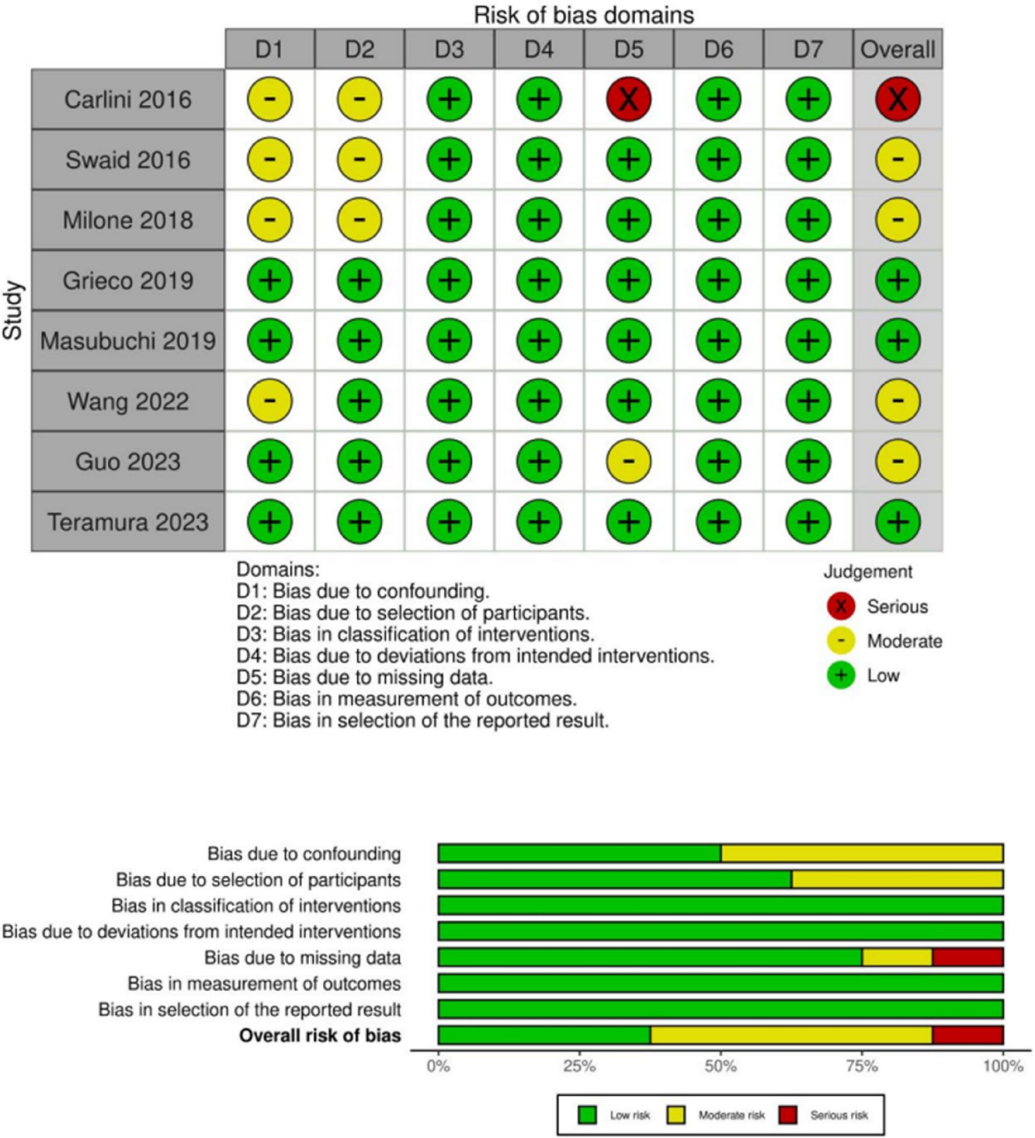
Risk of bias summary and graphical visualization of the included studies based on ROBINS-I-tool

Six of the included studies^29,31–35^ were retrospective and 2 studies^28,30^ were prospectively conducted. Propensity matching was performed in 4 studies.^31,32,34,35^ According to the ROBINS-I tool, the risk of bias was low to serious (Fig. 4). The most limiting factor was the lack of randomization in all included studies. The quality of evidence for the significant primary and secondary outcomes ranged between very low and moderate with respect to the GRADE criteria (Table S2).

## Discussion

The present study represents, to our knowledge, the first meta-analysis of pooled surgical outcomes of IA and EA in minimally-invasive left-sided colectomy for benign and malignant pathologies. We intentionally excluded studies of sigmoid colectomy and anterior rectal resections using the circular stapler because these cases represent a different anastomotic technique that is not applicable to left-sided colectomy extending to the proximal third of the sigmoid colon. The cumulative results of 8 included studies with 750 patients demonstrated a significant benefit of IA over EA in terms of overall morbidity, SSI, length of incision, time to first oral diet intake and time to first bowel movement while the surgical duration was significantly longer performing IA. At the same time, most of the important short-term outcomes were not significantly different, including anastomotic leak, ileus, severe postoperative morbidity (Clavien-Dindo > III), postoperative pain score, length of hospital stay, and specimen factors. These findings are consistent with some recently published studies of IA and EA in laparoscopic and robotic right colectomy.^13–15,36^ Minimally-invasive techniques including laparoscopic and laparoscopic-assisted colectomy have become the standard approach in colorectal surgery, demonstrating superior short-term recovery and equivalent oncologic outcomes compared to open surgery.^3,4,37–39^ Of note, none of the studies included in our analysis used a robotic platform for IA and EA. However, recent studies suggest that the robotic system is increasingly being used for left colectomy, with similar outcomes compared to laparoscopic resections.^40,41^ Minimally-invasive left-sided colectomy is technically more complex than right-sided colectomy because mobilization of both attached colonic ends and the splenic flexure is obligatory.^29^ Reconstruction of gastrointestinal continuity in minimally-invasive surgery is performed by either IA or EA. An advantage of IA is less bowel manipulation and exteriorization and consequently less mesenteric traction and twisting through a smaller abdominal incision.^10,42–44^ This could be very practical in obese patients with a shortened and thickened bowel mesentery and extensive subcutaneous tissue.^13,45^ This may explain why BMI was significantly higher in the IA group (29.5 versus 24.7, *p* = 0.05) in the study by Milone et al.^30^. Nevertheless, EA is still widely used because it is less technically demanding and allows hand-sewn enterotomy closure. Our meta-analysis demonstrated that IA offers several advantages compared to EA, while adverse outcomes are limited. The mean length of incision was significantly longer in EA compared to IA (9.1 cm versus 5.6 cm, *p* = 0.004). An obvious advantage of IA is the possibility to perform a smaller incision for specimen extraction regardless of the location of the anastomosis, whereas in EA the location of the incision or extraction is tied to the height of the planned anastomosis and sometimes the incision must be extended to create a tension-free anastomosis. In almost all studies, the Pfannenstiel incision was used as the site of extraction in IA as opposed to midline and off-midline laparotomies in the EA group. A recently published meta-analysis showed that the midline extraction site has a 16% incisional hernia rate compared to only 2.1% after the Pfannenstiel incision in minimally-invasive colorectal surgery.^46^ In addition, the Pfannenstiel incision is associated with less pain, better cosmetic results, and less wound infection.^47,48^ Only one study^31^ reported the incisional hernia rate, as most studies did not provide complete long-term follow-up information. In the study by Grieco et al.^31^ the rate of incisional hernias was 16.7% in the EA and only 2.8% in the IA cohort (*p* = 0.047), possibly due to the longer incision length and incision site in the EA group. In this study, the incision site in EA was located in the left subcostal region. Emile et al.^15^ also demonstrated a significantly higher incidence of incisional hernias after EA in right colectomy.

Length of hospital stay was similar between the two groups, although a trend toward earlier discharge was observed in the IA group. This could be attributed to the lower rate of overall complications, earlier resumption of gastrointestinal motility and oral feeding. Length of hospital stay is per se a topic of great variability, considering that studies from 5 different countries with different institutional perioperative care policies were included as a potential source of heterogeneity, despite adherence to recovery protocols. However, studies comparing IA with EA in laparoscopic right colectomy have reported inconsistent results regarding length of hospital stay.^13,14,49^ Interestingly, our subgroup analysis showed that the length of hospital stay was shorter in the IA group only in studies with a higher number of patients.^30,31,33^

Operative time was reported in 7 included studies^28–34^ and revealed a significant longer surgical duration in the IA group. This is line with some recently published meta-analysis comparing IA versus EA in minimally-invasive right colectomy.^13,50^ Interestingly, some authors stated that the practice of the more challenging IA at their institution was adapted to laparoscopic right colectomy years before left colectomy, which helped them to achieve a faster learning curve and equal operative time for both methods.^29^ Despite this successful transition, in our analysis the mean duration of surgery was approximately 18 min longer in the IA group as compared to EA.

Postoperative pain assessment was performed in 3 studies, but only 2 studies^32,33^ subdivided the pain score from day 0 to day 3 and 1 study^30^ provided an overall pain score. However, the reported results suggest no significant difference in postoperative pain on days 0 and 3 despite a longer incision in the EA group.

Interestingly, no difference in anastomotic leakage rate was observed, which may be partially explained by the fact that both IA and EA are performed by the same technical method using a stapling device in the majority of cases included. Indeed, it has been shown that stapled ileocolic anastomosis is associated with lower rates of anastomotic leakage compared to hand-sewn anastomosis.^51,52^ This observation may suggest that extensive bowel manipulation and traction does not affect anastomotic integrity, but rather negatively affects bowel contractility and peristalsis,^53^ as evidenced by significantly faster bowel recovery in IA. The rate of intra-abdominal infection and fluid collection was comparable in the IA and EA groups, which mitigates concerns about contamination of the abdominal cavity during bowel opening and anastomosis creation in IA. In this context, adequate peritoneal lavage,^32^ use of atraumatic intracorporeal bulldogs,^54^ and prophylactic antibiotic administration^55^ seem to prevent this complication. A recently published study suggested that EA after laparoscopic right colectomy is associated with a higher immune stress response (SSR), as indicated by significantly elevated interleukin-6 and C-reactive protein (CRP) levels on postoperative days 1, 3, and 5 in EA,^56^ which in turn may impair bowel recovery. In our meta-analysis, postoperative inflammatory markers are only mentioned by 3 authors.^32,33,35^ In these studies, bowel recovery outcomes^32,33^ and overall complications^35^ were in favor of IA despite significantly elevated CRP levels in the IA group.

Notably, wound infections were significantly higher in the EA group. This finding is consistent with data from minimally-invasive right colectomy studies.^13–15,49^ The reason for this observation is explained by the fact that the smaller incision in IA is only used for specimen extraction, in contrast to EA, where the usually larger mini-laparotomy site is also used for anastomosis creation of traction and trauma exposed bowel ends and thus carries the risk of potential bacterial contamination.

The number of harvested lymph nodes, specimen length, and the resections margin were similar in both groups, and furthermore, mid-term oncological data reported in two studies^28,33^ support comparable outcomes in IA and EA patients, with no increased risk of tumor recurrence in the IA group. Importantly, based on the provided data we were not able to perform a meta-analysis of the anastomosis type effect on conversion to laparotomy as only one study^30^ reported 21 conversions (IA *n* = 2 versus EA *n* = 19, *p* < 0.001) and the remaining three studies^28,29,32^ had no conversions in both groups.

Despite the novelty of our meta-analysis in the field of minimally- invasive colorectal surgery, the reported results have some considerable limitations, including the retrospective^29,31–35^ and mono-centric^28,29,32,33,35^ design in the majority of studies. In addition, none of the studies were from North America and a low mean BMI was observed in all studies. The sample sizes of the available studies were relatively small, with 5 studies analyzing ≤ 72 patients in their cohorts.^28,29,31,32,35^

When interpreting the results, the non-negligible effect of technical evolution during the long study period starting in 2004 must be considered. The short-term follow-up of 30 days in 5 studies^29–31,34,35^ did not allow evaluation of the some important outcome parameters including incisional hernia. A longer observation period could possibly show a persistent advantage of IA in the incisional hernia rate, as observed in comparable literature on right colectomy.^15,57^ Finally, the lack of randomization in all studies and potential selection bias led to a classification of "moderate" bias in 4 studies^29,30,33,34^ and "serious" risk of bias in one study.^28^ Therefore, the level of evidence for the important primary and secondary outcomes was very low to moderate considering the above mentioned limitations. Furthermore, interpretation of the data must take into account differences in institutional perioperative care policies, as well as differences in surgical experience and preference. Although the results provide a surrogate outcome advantage for IA, we cannot generally recommend this method as the "standard" approach for minimally-invasive left colectomy. Large randomized controlled trials with long follow-up data are needed to clarify the question of the most appropriate anastomotic technique in this setting.

## Conclusion

IA in minimally-invasive left colectomy for benign and malignant lesions proves to be a safe and feasible option despite technical challenges and a longer procedural duration. It was associated with less overall morbidity, less SSI, shorter incision length and faster postoperative gastrointestinal recovery compared to EA. At the same time, oncologic radicality and outcomes appear to be equivalent in both IA and EA groups. Large randomized controlled trials are needed to further validate these results.

### Supplementary Information

Below is the link to the electronic supplementary material.

Supplementary file1 (DOCX 66 kb)

Supplementary file2 (DOCX 15 kb)

Supplementary file3 (DOCX 30 kb)

## References

[1] Fowler DL, White SA (1991). Laparoscopy-assisted sigmoid resection. Surg Laparosc Endosc.

[2] Braga M, Frasson M, Zuliani W (2010). Randomized clinical trial of laparoscopic versus open left colonic resection. Br J Surg.

[3] Lacy AM, García-Valdecasas JC, Delgado S (2002). Laparoscopy-assisted colectomy versus open colectomy for treatment of non-metastatic colon cancer: a randomised trial. Lancet.

[4] McCombie AM, Frizelle F, Bagshaw PF (2018). The ALCCaS Trial: A Randomized Controlled Trial Comparing Quality of Life Following Laparoscopic Versus Open Colectomy for Colon Cancer. Dis Colon Rectum.

[5] Tjandra JJ, Chan MKY (2006). Systematic review on the short-term outcome of laparoscopic resection for colon and rectosigmoid cancer. Colorectal Dis.

[6] Veldkamp R, Kuhry E, Hop WCJ (2005). Laparoscopic surgery versus open surgery for colon cancer: short-term outcomes of a randomised trial. Lancet Oncol.

[7] Guller U, Jain N, Hervey S (2003). Laparoscopic vs open colectomy: outcomes comparison based on large nationwide databases. Arch Surg.

[8] Bagshaw PF, Allardyce RA, Frampton CM (2012). Long-term outcomes of the australasian randomized clinical trial comparing laparoscopic and conventional open surgical treatments for colon cancer: the Australasian Laparoscopic Colon Cancer Study trial. Ann Surg.

[9] Jayne DG, Thorpe HC, Copeland J (2010). Five-year follow-up of the Medical Research Council CLASICC trial of laparoscopically assisted versus open surgery for colorectal cancer. Br J Surg.

[10] Grams J, Tong W, Greenstein AJ, Salky B (2010). Comparison of intracorporeal versus extracorporeal anastomosis in laparoscopic-assisted hemicolectomy. Surg Endosc.

[11] Minjares RO, Dimas BA, Ghabra S (2020). Surgical resection for diverticulitis using robotic natural orifice intracorporeal anastomosis and transrectal extraction approach: the NICE procedure. J Robot Surg.

[12] van Oostendorp S, Elfrink A, Borstlap W (2017). Intracorporeal versus extracorporeal anastomosis in right hemicolectomy: a systematic review and meta-analysis. Surg Endosc.

[13] Zheng J-C, Zhao S, Chen W (2021). Comparison of intracorporeal and extracorporeal anastomosis and resection in right colectomy: a systematic review and meta-analysis. Langenbecks Arch Surg.

[14] Zhang H, Sun N, Fu Y, Zhao C (2021). Intracorporeal versus extracorporeal anastomosis in laparoscopic right colectomy: updated meta-analysis of randomized controlled trials. BJS Open.

[15] Emile SH, Elfeki H, Shalaby M (2019). Intracorporeal versus extracorporeal anastomosis in minimally invasive right colectomy: an updated systematic review and meta-analysis. Tech Coloproctol.

[16] Liang Y, Li L, Su Q (2022). Short-term outcomes of intracorporeal and extracorporeal anastomosis in robotic right colectomy: a systematic review and meta-analysis. Tech Coloproctol.

[17] Jamali FR, Soweid AM, Dimassi H (2008). Evaluating the degree of difficulty of laparoscopic colorectal surgery. Arch Surg.

[18] Han J, Min BS (2016). Laparoscopic-assisted radical left hemicolectomy for colon cancer. J Vis Surg.

[19] Moher D, Liberati A, Tetzlaff J (2009). Preferred reporting items for systematic reviews and meta-analyses: the PRISMA statement. PLoS Med.

[20] Cochrane Handbook for Systematic Reviews of Interventions. https://training.cochrane.org/handbook. Accessed 27 Feb 2022

[21] Dindo D, Demartines N, Clavien P-A (2004). Classification of surgical complications: a new proposal with evaluation in a cohort of 6336 patients and results of a survey. Ann Surg.

[22] Sterne JA, Hernán MA, Reeves BC (2016). ROBINS-I: a tool for assessing risk of bias in non-randomised studies of interventions. BMJ.

[23] Guyatt GH, Oxman AD, Kunz R (2011). GRADE guidelines: 7. Rating the quality of evidence–inconsistency. J Clin Epidemiol.

[24] Malmivaara A (2015). Methodological considerations of the GRADE method. Ann Med.

[25] Hozo SP, Djulbegovic B, Hozo I (2005). Estimating the mean and variance from the median, range, and the size of a sample. BMC Med Res Methodol.

[26] Luo D, Wan X, Liu J, Tong T (2018). Optimally estimating the sample mean from the sample size, median, mid-range, and/or mid-quartile range. Stat Methods Med Res.

[27] Higgins JPT, Thompson SG, Deeks JJ, Altman DG (2003). Measuring inconsistency in meta-analyses. BMJ.

[28] Carlini M, Spoletini D, Castaldi F (2016). Laparoscopic resection of splenic flexure tumors. Updates Surg.

[29] Swaid F, Sroka G, Madi H (2016). Totally laparoscopic versus laparoscopic-assisted left colectomy for cancer: a retrospective review. Surg Endosc.

[30] Milone M, Angelini P, Berardi G (2018). Intracorporeal versus extracorporeal anastomosis after laparoscopic left colectomy for splenic flexure cancer: results from a multi-institutional audit on 181 consecutive patients. Surg Endosc.

[31] Grieco M, Cassini D, Spoletini D (2019). Intracorporeal Versus Extracorporeal Anastomosis for Laparoscopic Resection of the Splenic Flexure Colon Cancer: A Multicenter Propensity Score Analysis. Surg Laparosc Endosc Percutan Tech.

[32] Masubuchi S, Okuda J, Hamamoto H, et al (2019) Intracorporeal Versus Extracorporeal Anastomosis in Laparoscopic Left Colectomy Forleft-Side Colon Cancer: A Retrospective Study. Clinics In Surgery 4:

[33] Wang L-M, Jong B-K, Liao C-K (2022). Comparison of short-term and medium-term outcomes between intracorporeal anastomosis and extracorporeal anastomosis for laparoscopic left hemicolectomy. World J Surg Oncol.

[34] Guo Y, Li K, He L (2023). Surgical site infection after intracorporeal and extracorporeal anastomosis in laparoscopic left colectomy for colon cancer: a multicenter propensity score-matched cohort study. Surg Endosc.

[35] Teramura K, Kitaguchi D, Matsuoka H (2023). Short-term outcomes following intracorporeal versus extracorporeal anastomosis after laparoscopic right and left-sided colectomy: a propensity score-matched study. Int J Surg.

[36] Ishizuka M, Shibuya N, Takagi K (2022). Postoperative Complications Associated With Intra- Versus Extracorporeal Anastomosis for Laparoscopic Right Colectomy. Am Surg.

[37] Juo Y-Y, Hyder O, Haider AH (2014). Is minimally invasive colon resection better than traditional approaches?: First comprehensive national examination with propensity score matching. JAMA Surg.

[38] Hazebroek EJ, Color Study Group (2002). COLOR: a randomized clinical trial comparing laparoscopic and open resection for colon cancer. Surg Endosc.

[39] Nelson H, Sargent DJ, Clinical Outcomes of Surgical Therapy Study Group (2004). A comparison of laparoscopically assisted and open colectomy for colon cancer. N Engl J Med.

[40] Solaini L, Giuliani G, Cavaliere D (2023). Robotic versus laparoscopic left colectomy: a propensity score matched analysis from a bi-centric experience. J Robot Surg.

[41] Serra-Aracil X, Mora-Lopez L, Gomez-Torres I (2023). Laparoscopic and robotic intracorporeal resection and end-to-end anastomosis in left colectomy: a prospective cohort study - stage 2a IDEAL framework for evaluating surgical innovation. Langenbecks Arch Surg.

[42] Jian-Cheng T, Shu-Sheng W, Bo Z (2016). Total laparoscopic right hemicolectomy with 3-step stapled intracorporeal isoperistaltic ileocolic anastomosis for colon cancer: An evaluation of short-term outcomes. Medicine (Baltimore).

[43] Hanna MH, Hwang GS, Phelan MJ (2016). Laparoscopic right hemicolectomy: short- and long-term outcomes of intracorporeal versus extracorporeal anastomosis. Surg Endosc.

[44] Hellan M, Anderson C, Pigazzi A (2009). Extracorporeal versus intracorporeal anastomosis for laparoscopic right hemicolectomy. JSLS.

[45] Blumberg D (2009). Laparoscopic colectomy performed using a completely intracorporeal technique is associated with similar outcome in obese and thin patients. Surg Laparosc Endosc Percutan Tech.

[46] den Hartog FPJ, van Egmond S, Poelman MM (2022). The incidence of extraction site incisional hernia after minimally invasive colorectal surgery: a systematic review and meta-analysis. Colorectal Dis.

[47] Orcutt ST, Balentine CJ, Marshall CL (2012). Use of a Pfannenstiel incision in minimally invasive colorectal cancer surgery is associated with a lower risk of wound complications. Tech Coloproctol.

[48] Kisielinski K, Conze J, Murken AH (2004). The Pfannenstiel or so called “bikini cut”: still effective more than 100 years after first description. Hernia.

[49] Aiolfi A, Bona D, Guerrazzi G (2020). Intracorporeal Versus Extracorporeal Anastomosis in Laparoscopic Right Colectomy: An Updated Systematic Review and Cumulative Meta-Analysis. J Laparoendosc Adv Surg Tech A.

[50] Zhang T, Sun Y, Mao W (2023). Meta-analysis of randomized controlled trials comparing intracorporeal versus extracorporeal anastomosis in minimally invasive right hemicolectomy: upgrading the level of evidence. Int J Colorectal Dis.

[51] Choy PYG, Bissett IP, Docherty JG, et al (2011) Stapled versus handsewn methods for ileocolic anastomoses. Cochrane Database Syst Rev CD004320. 10.1002/14651858.CD004320.pub3 10.1002/14651858.CD004320.pub3 21901690

[52] Luglio G, Corcione F (2019). Stapled versus handsewn methods for ileocolic anastomoses. Tech Coloproctol.

[53] Bollo J, Turrado V, Rabal A (2020). Randomized clinical trial of intracorporeal versus extracorporeal anastomosis in laparoscopic right colectomy (IEA trial). Br J Surg.

[54] Chang K, Fakhoury M, Barnajian M (2013). Laparoscopic right colon resection with intracorporeal anastomosis. Surg Endosc.

[55] Ho VP, Barie PS, Stein SL (2011). Antibiotic regimen and the timing of prophylaxis are important for reducing surgical site infection after elective abdominal colorectal surgery. Surg Infect (Larchmt).

[56] Mari GM, Crippa J, Costanzi ATM (2018). Intracorporeal Anastomosis Reduces Surgical Stress Response in Laparoscopic Right Hemicolectomy: A Prospective Randomized Trial. Surg Laparosc Endosc Percutan Tech.

[57] Selznick S, Levy J, Bogdan R-M (2023). Laparoscopic right colectomies with intracorporeal compared to extracorporeal anastomotic techniques are associated with reduced post-operative incisional hernias. Surg Endosc.

