# Supplementary material for: Intracorporeal Versus Extracorporeal Colo-colic Anastomosis in Minimally-invasive Left Colectomy: a Systematic Review and Meta-analysis
Source: J Gastrointest Surg. 2023 Sep 12;27(12):3024–37. doi: 10.1007/s11605-023-05827-1 (PMC10837220; doi:10.1007/s11605-023-05827-1)
Supplement: Supplementary file 1 — Supplementary file1 (DOCX 66 kb) [file 11605_2023_5827_MOESM1_ESM.docx]

**Table S1** Subgroup analysis length of hospital stay

|  |  |  | Hospitalization time |  |  |  |
| --- | --- | --- | --- | --- | --- | --- |
| Subgroup | No. of included studies | No. of included patients | SMD [95 % CI] | p-value | I² (%) | Chi^2^ (p-value) |
| Study size |  |  |  |  |  |  |
| < 62 cases | 4 [28, 29, 32, 35] | 155 | -0.22 [-1.21-0.78] | 0.67 | 88 | < 0.0001 |
| ≥ 62 cases | 3 [30, 31, 33] | 370 | -0.53 [-0.86 to -0.20] | 0.002 | 55 | 0.11 |
| Study bias |  |  |  |  |  |  |
| Low | 3 [31, 32, 35] | 155 | -0.33 [-1.21-0.55] | 0.46 | 86 | 0.0010 |
| Moderate-High | 4 [28–30, 33] | 370 | -045 [-1.02-0.12] | 0.12 | 82 | 0.0010 |
| Multi vs. single-center |  |  |  |  |  |  |
| Multi-center | 2 [30, 31] | 253 | -0.48 [-0.98-0.01] | 0.06 | 69 | 0.07 |
| Single-center | 5 [28, 29, 32, 33, 35] | 272 | -0.33 [-1.03-0.37] | 0.36 | 85 | < 0.0001 |
| PSM |  |  |  |  |  |  |
| Yes | 3 [31, 32, 35] | 155 | -0.33 [-1.21-0.55] | 0.46 | 86 | 0.0010 |
| No | 4 [28–30, 33] | 370 | -0.45 [-1.02-0.12] | 0.12 | 82 | 0.0010 |
| Study origin |  |  |  |  |  |  |
| Europe | 4 [28–31] | 325 | -0.47 [-1.09-0.15] | 0.14 | 82 | 0.0007 |
| Asia | 3 [32, 33, 35] | 200 | -0.30 [-1.11-0.51] | 0.47 | 85 | 0.001 |
|  |  |  |  |  |  |  |

OR: odds ratio, SMD: standardized mean difference, PSM: propensity score matching
