# Supplementary material for: Intracorporeal Versus Extracorporeal Colo-colic Anastomosis in Minimally-invasive Left Colectomy: a Systematic Review and Meta-analysis
Source: J Gastrointest Surg. 2023 Sep 12;27(12):3024–37. doi: 10.1007/s11605-023-05827-1 (PMC10837220; doi:10.1007/s11605-023-05827-1)
Supplement: Supplementary file 2 — Supplementary file2 (DOCX 15 kb) [file 11605_2023_5827_MOESM2_ESM.docx]

Table S2 The GRADE Certainty assessment for the significant primary and secondary outcomes

| Outcomes | No. of studies | **No. of included patients** | | SMD/OR [95 % CI] | **Quality assessment** | | | | | Quality |
| --- | --- | --- | --- | --- | --- | --- | --- | --- | --- | --- |
|  |  | IA | EA |  | Risk of bias^a^ | Inconsistency | Indirectness | Imprecision | Publication bias |  |
| Overall morbidity | 8 [28–35] | 335 | 415 | 0.40 [0.26-0.61] | Not serious | Not serious | No indirectness | No imprecision | NA | Moderate |
| SSI | 8 [28–35] | 335 | 415 | 0.27 [0.12-0.61] | Not serious | Not serious | No indirectness | No imprecision | NA | Moderate |
| Incision length | 5 [29–32] | 181 | 164 | -2.51 [-4.21 to -0.81] | Not serious | Very serious | No indirectness | No imprecision | NA | Very low |
| First solid diet | 3 [31–33] | 96 | 133 | -0.49 [-0.76 to -0.22] | Not serious | Not serious | No indirectness | No imprecision | NA | Moderate |
| Fist stool passage | 4 [30–33] | 188 | 222 | -0.40 [-0.71 to -0.09] | Not serious | Serious | No indirectness | No imprecision | NA | Low |
| Operative duration | 7 [28–34] | 314 | 393 | 0.36 [0.14-0.59] | Not serious | Not serious | No indirectness | No imprecision | NA | Moderate |

OR: odds ratio, SMD: standardized mean difference, IA; intracorporeal anastomosis, EA: extracorporeal anastomosis, SSI: surgical site infection ^a^ Risk of bias assessed using the ROBINS-I tool.
